# Deficiency of the Ribosomal Protein uL5 Leads to Significant Rearrangements of the Transcriptional and Translational Landscapes in Mammalian Cells

**DOI:** 10.3390/ijms222413485

**Published:** 2021-12-15

**Authors:** Elena S. Babaylova, Alexander V. Gopanenko, Alexey E. Tupikin, Marsel R. Kabilov, Alexey A. Malygin, Galina G. Karpova

**Affiliations:** Institute of Chemical Biology and Fundamental Medicine, Siberian Branch of the Russian Academy of Sciences, Prospekt Lavrentieva 8, 630090 Novosibirsk, Russia; lalena2005@ngs.ru (E.S.B.); alexandr.gopanenko@yandex.ru (A.V.G.); alenare@niboch.nsc.ru (A.E.T.); kabilov@niboch.nsc.ru (M.R.K.); malygin@niboch.nsc.ru (A.A.M.)

**Keywords:** HEK293T cells, knockdown of ribosomal protein uL5, next-generation sequencing, RNA-seq, polysome profiling, differential gene expression, uL5-dependent genes, uL5-related processes

## Abstract

Protein uL5 (formerly called L11) is an integral component of the large (60S) subunit of the human ribosome, and its deficiency in cells leads to the impaired biogenesis of 60S subunits. Using RNA interference, we reduced the level of uL5 in HEK293T cells by three times, which caused an almost proportional decrease in the content of the fraction corresponding to 80S ribosomes, without a noticeable diminution in the level of polysomes. By RNA sequencing of uL5-deficient and control cell samples, which were those of total mRNA and mRNA from the polysome fraction, we identified hundreds of differentially expressed genes (DEGs) at the transcriptome and translatome levels and revealed dozens of genes with altered translational efficiency (GATEs). Transcriptionally up-regulated DEGs were mainly associated with rRNA processing, pre-mRNA splicing, translation and DNA repair, while down-regulated DEGs were genes of membrane proteins; the type of regulation depended on the GC content in the 3′ untranslated regions of DEG mRNAs. The belonging of GATEs to up-regulated and down-regulated ones was determined by the coding sequence length of their mRNAs. Our findings suggest that the effects observed in uL5-deficient cells result from an insufficiency of translationally active ribosomes caused by a deficiency of 60S subunits.

## 1. Introduction

The final stage in gene expression is translation, the process of synthesizing proteins from amino acid residues in accordance with genetic information embedded in mRNAs. Consequently, the cellular repertoire of protein-coding gene products directly depends on the proper operation of the translation machinery. Disorders in the functioning of this machinery affect the composition of the cellular proteome, and therefore, when they arise, the cell faces the task of adjusting the translation mechanism by enhancing and/or weakening the expression of the appropriate genes. Ribosomes, being the main components of the protein-synthesizing system, along with many other factors, play a regulatory role in the translation process (e.g., see [1,2]). In the mammalian 80S ribosome, the large (60S) subunit and the small (40S) subunit together contain 80 proteins and 4 rRNAs. The pathological and stressful conditions of development can cause a decrease in the level of some ribosomal proteins, leading to their deficiency during the assembly of the 60S and/or 40S subunits. In addition, mutations can appear in the genes of ribosomal proteins, resulting in the synthesis of the corresponding aberrant ones that should either, being incorporated into the ribosomal subunits, distort their structure, making it unfavorable for translation, or be unable to participate in the assembly of the subunits at all. A deficiency of functionally active ribosomal proteins, regardless of the cause of its occurrence, ultimately leads to disruptions in the biogenesis of ribosomal subunits, translation and those numerous processes in which the proteins outside the ribosome play an important role, performing their extraribosomal functions. To date, a lot of data has been accumulated, indicating the existence of a relationship between the above disorders and diseases, such as ribosomopathies and various forms of cancer [3,4,5,6].

One of the ribosomal proteins that play an essential role in ribosome biogenesis and whose deficiency is directly related to carcinogenesis is the ribosomal protein uL5 (formerly known as L11). During the assembly of the 60S subunits, uL5, together with the ribosomal protein uL18 (formerly known as L5), is recruited to the 60S pre-subunits as a preformed subcomplex with 5S rRNA (5S RNP) bound to the assembly factors RRS1 and BXDC1 and to the tumor suppressor protein GLTSCR2 that is required for the integration of 5S RNP into the 60S pre-subunits [6]. Notably, the 5S RNP, being in an unstable conformation, initially associates with the very early nucleolar 60S pre-subunits and is stabilized by 180° rotations at a later stage of maturation of 60S pre-subunits, which makes this stage a checkpoint for the assembly of 60S subunits [7].

Defects in the biogenesis of 60S subunits lead to increased levels of non-ribosome-bound 5S RNP in the nucleoplasm, where uL5 becomes implicated in the coordination of the levels and activities of cellular oncoproteins. In particular, uL5 has been identified as a regulator of the c-Myc oncoprotein, which is responsible for the activation of the expression of many genes involved in cell proliferation and ribosome biogenesis; c-Myc activities are enhanced with a decrease in the uL5 level [8]. When uL5 is in excess, it binds to the 3′ untranslated region (UTR) of c-Myc mRNA and promotes its degradation by recruiting a specific miRNA to the 3′ UTR, while the knockdown of the protein dramatically increases the level of c-Myc due to the stabilization of c-Myc mRNA [9]. It is noteworthy that uL5 interacts with c-Myc mRNA together with the ribosomal protein uL18, its aforementioned 5S rRNA binding partner, and thus, both proteins destabilize this mRNA by being part of 5S RNP [10].

As a component of the 5S RNP, the ribosomal protein uL5 binds to another oncogene, MDM2, and regulates its activity [11,12]. This binding prevents the MDM2-mediated ubiquitination and degradation of the tumor suppressor p53 [13]. It has been thought that the reduction of uL5 levels should result in MDM2 activation and, accordingly, in a decrease in p53 level [11,12]. However, it has turned out that uL5 deficiency leads to an increase in p53 activity in both zebrafish [14] and human lung fibroblast cells [15,16]. Along with this, there has also been a strong suppression of the cell cycle progression due to the inhibition of the global translation capacity, caused by a ribosome deficiency provoked by the defective biogenesis of 60S subunits [14,16]. The haploinsufficiency of functional uL5, resulting from mutations in the RPL11 gene, has also been identified as a common cause of Diamond-Blackfan anemia (DBA) [17,18]; to date, 32 mutations in this gene have been described that occur in patients with DBA [19].

To get an idea of the general global changes in the transcriptomic and translatomic landscapes of mammalian cells occurring with a decrease in uL5 levels, we applied high-throughput RNA sequencing (RNA-seq) and polysome profiling, followed by RNA-seq to HEK293T cells, with a deficiency of uL5 caused by their transfection with appropriate specific siRNAs. By analyzing the sequencing data, we revealed sets of differentially expressed genes at transcriptional and translational levels, (t)DEGs and (p)DEGs, respectively, between cells with the reduced content of uL5 and control cells that were treated with non-targeting siRNA, and we identified genes with an altered translational efficiency (GATEs). This allowed us to demonstrate the impact of the ribosomal protein uL5 deficiency on the expression of particular genes at the levels of transcription and translation and to reveal the specific features of the mRNAs of (t)DEGs and GATEs, which determine the occurrence of these genes in the respective sets of up-regulated and down-regulated ones. The results obtained argue that all the changes in the expression levels and translational efficiencies of genes found in uL5-deficient cells, as compared to control ones, are a consequence of a decrease in the number of ribosomes due to the impaired biogenesis of 60S subunits.

## 2. Results

### 2.1. Characterization of uL5-Deficient Cells

**Figure 1:**
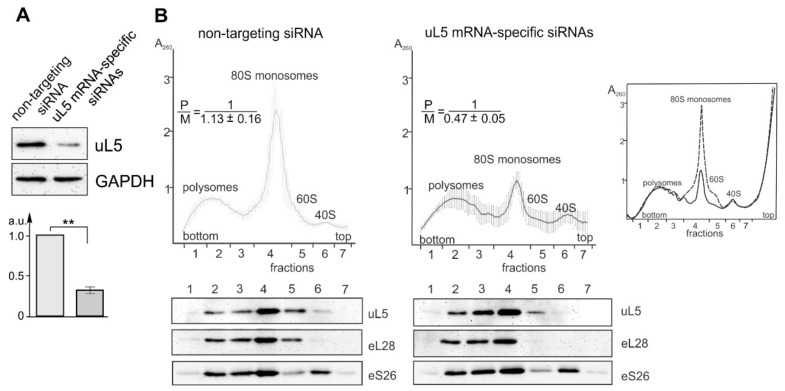
The characterization of HEK293T cells knocked down of ribosomal protein uL5. (**A**) Western blot analysis of the levels of uL5 and GAPDH (as a reference) in cells transfected with uL5-specific siRNAs and non-targeting siRNA. The diagram shows triplicate data as the mean of arbitrary units (A.U.) ± SEM (** *p* < 0.01, calculated using a Mann–Whitney test). (**B**) Polysome profiles obtained by the sucrose density gradient centrifugation of the lysates of cells transfected with non-targeting siRNA (left) and uL5 mRNA-specific siRNAs (middle). The peaks of 60S and 40S subunitsand 80S monosomes and polysomes are marked. The averaged polysome profiles are derived from flow cell measurements and shown as curves drawn through 100 points with error bars, which correspond to the average absorbance at 260 nm ± SD of 4 replicates. P/M means the ratio between the peaks of polysomes and 80S monosomes calculated from 4 replicates. Below each profile, data from the Western blot analysis of the sucrose gradient fractions for the presence of uL5 and ribosomal proteins eL28 and eS26 (as references) carried out using specific antibodies are presented. A superposition of polysome profiles obtained from uL5 knocked down cells (solid line) and cells treated with non-targeting siRNA (dashed line) is shown for one of the replicates of each cell sample in a separate box on the right.

The deficiency of ribosomal protein uL5 in HEK293T cells was achieved using the RNA interference approach. To this end, cells grown in Petri dishes to 70% confluence were transfected with siRNAs specific for the mRNA of uL5 or non-targeting siRNA as a control. The MTT test revealed only minor differences in the viability of cells transfected with uL5 mRNA-specific siRNAs after culturing for 48 h compared to cells transfected with non-targeting siRNA (Figure S1). Time-course changes in the levels of ribosomal protein uL5 and its mRNA in the transfected cells were determined by Western blotting and RT-qPCR, respectively. It was shown that the level of uL5 mRNA in cells transfected with specific siRNAs dropped by 7 times in 24 h compared to that in untreated cells and remained unchanged for the next 24 h (Figure S2A), while the level of uL5 decreased more slowly and became reduced by 3 times after 48 h (Figure 1A and Figure S2B). No significant time-course changes in the levels of uL5 and its mRNA were observed in cells treated with non-targeting siRNA (Figure S2A,B). Thus, the duration of cell cultivation after transfection for subsequent harvesting and lysis was chosen to be 2 days.

Polysome profiles obtained with uL5-deficient and control cells were significantly different. Although the peaks corresponding to polysomes were almost the same ones, the peak of 80S monosomes, which overlaps with the peak of 60S subunits, was much lower in uL5-deficient cells (Figure 1B). To compare the contents of the ribosomal protein uL5 and the reference proteins eS26 and eL28, specific for 40S and 60S ribosomal subunits, respectively, in the 80S monosome and polysome fractions of the profiles, total protein from these fractions was analyzed by Western blotting using appropriate antibodies (Figure 1B). One can see that uL5 was present in the peaks of polysomes and 80S monosomes in the same proportion as the reference proteins eS26 and eL28, which indicates that uL5 is an indispensable part of the functionally active 60S ribosomal subunit. However, a decrease in the height of the peak common for 80S monosomes and 60S subunits in the profile of uL5 knocked down cells should primarily reflect a significant deficiency of mature 60S subunits, which, accordingly, leads to a decrease in the level of 80S monosomes. Indeed, the densitometric analysis of Western blot antibody responses shown in Figure 1B revealed that the 60S/40S ratio in uL5-deficient cells was more than halved compared to that in control cells. (Figure S3A). Noteworthy, the peak height of 40S subunits did not increase with a decrease in that of 80S monosomes, but remained the same as in the polysome profile from control cells (Figure 1B), indicating a turnover of 40S subunits in cells with a deficiency of 60S subunits, similar to that previously shown for yeasts [20]. This was also confirmed by the relative contents of proteins eS26, uL5, and eL28 in the total lysates of uL5-deficient and control cells. (Figure S3B).

### 2.2. RNA-Seq Data Analysis with HEK293T Cells Knocked down of uL5

**Figure 2:**
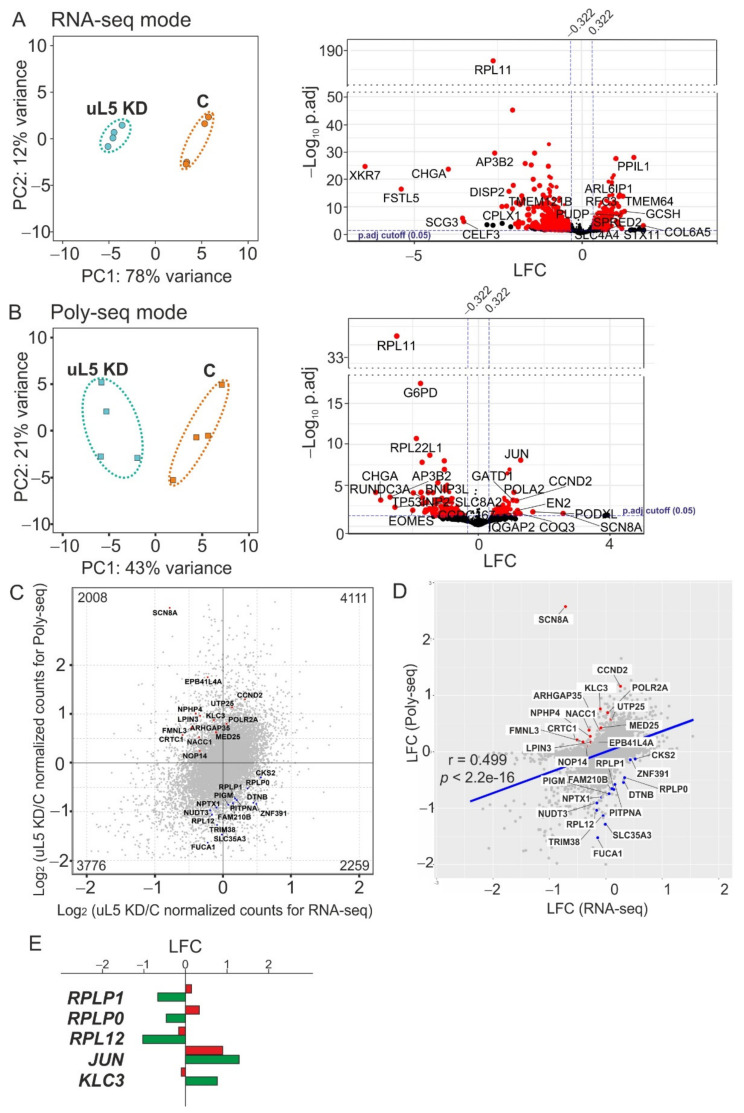
The general overview of data gained for uL5-deficient HEK293T cells in four biological replicates using the RNA-seq and polysome profiling followed by RNA-seq assays. (**A**) Analysis of RNA-seq data. Left panel, principal components analysis (PCA) data plot based on those obtained for total RNA samples from uL5 knocked down cells (uL5 KD) and control, non-targeting siRNA-treated cells (**C**). Right panel, a volcano plot exposing the results of the differential gene expression analysis using DESeq2, where each dot is a single gene plotted according to its shrunken Log2 Fold Change (LFC) and the negative logarithm of the *p*-value adjusted (p.adj). Genes with cutoffs of p.adj < 0.05 and shrunken LFC absolute values > 0.322 ((t)DEGs) are displayed in red (representative (t)DEGs are signed by HGNC symbol identifiers), and remaining genes are shown in black. The blue dashed lines indicate the threshold LFC values: 0.322 and −0.322, which correspond to 25% changes in gene expression. (**B**) Analysis of polysome profiling followed by RNA-seq (Poly-seq) data. The designations in the panels presenting data on the identification of differentially expressed genes at the translation level ((p)DEGs) are the same as in panels A. (**C**) Differential gene expression analysis for the identification of genes with altered translational efficiency (GATEs). Scatter plot showing the analysis data, where GATEs are marked with red and blue dots corresponding to genes with increased and decreased translational efficiencies, respectively. The numbers of genes in each quadrant deduced from a comparative analysis of the RNA-seq data and the Poly-seq data are shown. (**D**) The analysis of the correlation between the sets of (t)DEGs and (p)DEGs. The regression line is shown in blue; GATEs are marked with red and blue dots, as above. (**E**) Relative quantities of mRNAs for several representative (t)DEGs and (p)DEGs found using DESeq2 in RNA-seq and Poly-seq modes, shown by red and green columns, respectively.

RNA-seq assay was performed with samples of HEK293T cells treated with either specific siRNAs against uL5 mRNA or non-targeting siRNA and of their respective polysome fractions from four biological replicates. The total RNA from these cell samples after two days of transfection with the above siRNAs and the RNA from the polysome fractions were extracted using TRIzol and subjected to polyA selection. From the resulting RNA samples, the DNA libraries were prepared with their subsequent next generation sequencing (NGS). The basic characteristics of the DNA libraries are presented in Table S1. The principal component analysis (PCA) evaluation of the NGS data showed a high degree of clustering between biological replicates (Figure 2A,B, left panels). This implied that the data obtained were of sufficient quality for use in further downstream calculations, although the clustering in the analysis of the RNA-seq data was noticeably better than in that of the polysome profiling followed by RNA-seq ones. Filtered and quality checked raw reads were mapped to the reference human genome, where they predominantly fell into the regions of genes corresponding to protein-coding transcripts (Table S1).

The analysis of differential gene expression performed separately with the RNA-seq data and the polysome profiling followed by RNA-seq data using the DESeq2 package revealed groups of genes whose expression was altered at the transcriptional and translational levels, respectively, in uL5-deficient cells compared to cells treated with control siRNA (Table S2). Applying the cutoff parameters of the *p*-value adjusted (p.adj) and the absolute value of the shrunken Log2 Fold Change (LFC) to these two gene groups, we accordingly distinguished the sets of statistically significant transcriptionally and translationally differentially expressed genes, named above as (t)DEGs and (p)DEGs, respectively (Figure 2A,B, right panels and Tables S3 and S4). In these terms, (t)DEGs were genes whose total mRNA content was altered in cells with the reduced level of uL5, while (p)DEGs were genes whose mRNA content was changed in the polysomes. The set of (t)DEGs included 836 down-regulated and 699 up-regulated genes, while the set of (p)DEGs consisted of 127 and 95 such genes, respectively. The analysis of differential gene expression applied to the RNA-seq and polysome profiling followed by RNA-seq data simultaneously made it possible to reveal the set of 28 genes with altered translation efficiency, named above as GATEs, which included both up-regulated GATEs and down-regulated ones (Figure 2C–E and Table S5). By alterations in translation efficiency of genes in uL5-deficient cells compared to control ones, we here imply changes in levels of their mRNAs in polysomes relative to changes in those in the transcriptome. Accordingly, up-regulated and down-regulated GATEs are genes with increased and decreased relative levels of the encoded mRNAs in polysomes.

It should be noted that one reason for the moderate correlation of the (t)DEG and (p)DEG sets with each other (Figure 2D) could be related to some differences in the qualities of the RNA-seq data and the Poly-seq data mentioned above. As a result, genes that went through the p.adj and LFC cutoffs when analyzing the RNA-seq data could not undergo the same cutoffs when analyzing the polysome profiling followed by RNA-seq data, and vice versa. Meanwhile, a comparative analysis of the RNA-seq data and the Poly-seq data presented in Figure 2C showed that the numbers of genes in quadrants 2 and 4 differed, although not very much. Besides, the mean of the distribution of LFC residuals calculated from the LFC values estimated from the RNA-seq data and the Poly-seq data was nonzero (Figure S4). All this meant that (p)DEGs were real and not controlled by (t)DEGs.

### 2.3. Genes Depending on the Level of uL5

The majority of down-regulated (t)DEGs encoded proteins involved in the organization of the extracellular matrix, extracellular interactions and integrins. These were, for example, *LTBP2*, *COL5A1*, *COL7A1*, *SERPINH1*, *SPARC*, *LAMA5*, *JAM3*, *FURIN*, *COL4A1*, *LAMB2*, *LTBP4*, *ITGA3*, *LTBP3*, *AGRN*, *COL3A1*, *NCAM1*, *COL4A5* and *BSG* (Table S3). In contrast, the set of up-regulated (t)DEGs consisted of housekeeping genes encoding a large group of cytosolic and mitochondrial ribosomal proteins (e.g., *RPL35A*, *RPS13*, *MRPL43*, *RPL36*, *MRPL13*, *RPL35*, *MRPL32*, *RPS15A*, *MRPL34*, *RPS7* and *MRPS10*) and translation factors (e.g., *EIF2A*, *EIF3B*, *EIF4H*, *EIF3J* and *EIF2S1*) (Table S3). In addition, this (t)DEG set also contained genes encoding proteins implicated in splicing (e.g., *SF3B1*, *SNRPC*, *SNRPA1*, *SF3B6*, *HNRNPC* and *HNRNPU*) and DNA repair (e.g., *XRCC3*, *XRCC2*, *NEIL3*, *CHEK2* and *MNAT1*), as well as kinases (*CDK2*, *MAPK6* and *MAPK8*) and transcription factors (*ATF3*, *ELK4* and *JUN*).

Notably, among the (p)DEGs (Table S4), there were mainly genes identified as (t)DEGs (Table S3), although genes specific only to the (p)DEG set were also found (e.g., NUDT3, RPL12, PPP2R5B, TAB1, POLA2 and CCND2). The proteins encoded by these (p)DEGs turned out to be players in cellular events, such as DNA replication (POLA2) and signal transduction through the regulation of activities of the CDK (CCND2) and MAPK (TAB1) family kinases and protein phosphatase 2A (PPP2R5B), as well as the metabolism of inositol phosphate (NUDT3).

The genes revealed as GATEs (Table S5) encoded proteins with very different functions. The set of 14 up-regulated GATEs contained the genes for the kinesin light chain (*KLC3*), a component of the ribosomal small subunit processome (*UTP25*), a subunit of RNA polymerase II (*POLR2A*), transcriptional repressor (*NACC1*), transcriptional coactivator (*CREB1*) and several others. The 14 down-regulated GATEs included genes of ribosomal proteins that are integral components of the 60S subunit P stalk (*RPLP0*, *RPLP1* and *RPL12*), a component of the dystrophin-associated protein complex (*DTNB*), a lysosomal enzyme involved in the degradation of glycolipids (*FUCA1*) and others.

**Figure 3:**
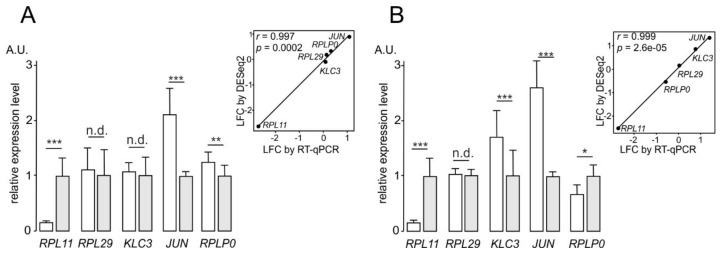
Validation of the results of the differential gene expression analyses with the RNA-seq and polysome profiling followed by RNA-seq (Poly-seq) data by RT-qPCR. The relative levels of mRNAs for the group of selected DEGs and reference genes found in total RNA samples (**A**) and in RNA samples from the respective polysome fractions (**B**) from uL5 knocked down HEK293T cells and control cells. The data are presented as the mean of arbitrary units (A.U.) from three or more replicates ± SEM (* *p* < 0.05, ** *p* < 0.01, *** *p* < 0.001 (Mann–Whitney test), n.d., no difference), displayed as dark and light columns for uL5 knocked down and control cells, respectively. Graphs showing the correlation between the RNA-seq and Poly-seq data and the RT-qPCR data are presented in boxes.

To validate the results of analyses of differential gene expression between cells knocked down of uL5 and control cells performed with the RNA-seq and polysome profiling followed by RNA-seq data, we carried out RT-qPCR analysis for a representative group of selected GATEs, as well as for *RPL11* and *RPL29* as references. The values of changes in the expression of these genes at the levels of transcription and translation, estimated by RT-qPCR, correlated with the respective values obtained using the above analyses utilizing the NGS data (Figure 3).

### 2.4. Cellular Processes Associated with (t)DEGs and GATEs

**Figure 4:**
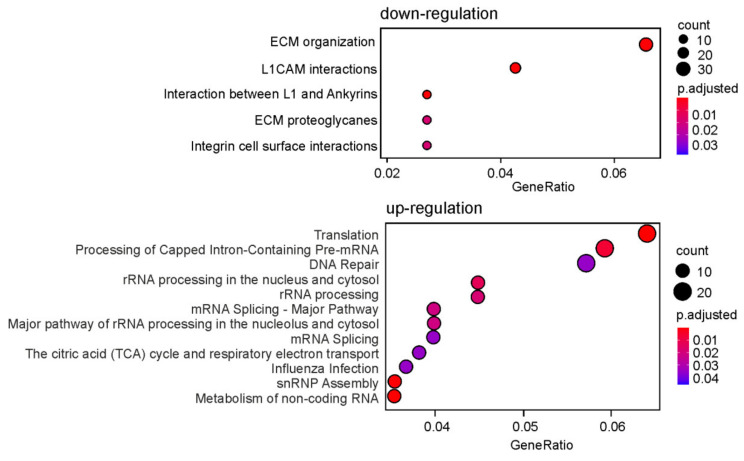
Dotplot enrichment maps showing cellular pathways associated with (t)DEGs whose activity is down-regulated (only the top 5 are shown) or up-regulated (only the top 12 are shown) in HEK293T cells knocked down of uL5. The colors of the points depend on p.adj values, and their sizes are determined by the numbers of (t)DEGs associated with the corresponding pathways in the Gene Ontology terms (color and dot size keys are shown to the right of the panels).

To identify the cellular processes associated with down-regulated and up-regulated (t)DEGs, the respective (t)DEG sets were analyzed using the ReactomePA package. We found that down-regulated (t)DEGs were mainly related to interactions of the L1 family of cell adhesion molecules (L1CAM), extracellular matrix (ECM) proteoglycans and membrane proteins, such as ankyrins and laminins (Figure 4, Table S6). The up-regulated (t)DEGs were involved in the basic cellular pathways, predominantly linked to DNA repair, rRNA processing, pre-mRNA splicing, translation and some others (Figure 4, Table S7).

No statistically significant enrichment was found for 28 GATEs (Table S5) in any cellular pathway, and no processes associated with them were revealed. Nevertheless, it should be noted that the set of 14 down-regulated GATEs, in addition to the three aforementioned ribosomal protein genes, contained genes involved in various events, including the regulation of the mitotic cell cycle (*CKS2*), lipid metabolic (*PITPNA*) and glycoside catabolic (*FUCA1*) processes, synaptic signaling (*DTNB*), cell–cell signaling (*NUDT3*) and several other. As for the 14 up-regulated GATEs, among them there were genes implicated in DNA-templated transcription (*POLR2A*) and its regulation (*NACC1* and *CRTC1*), positive regulation of protein phosphorylation (*CCND2*), pre-rRNA processing (*UTP25* and *NOP14*) and others.

### 2.5. The Deficiency of uL5 Affects the Cellular Level of mRNAs Depending on the Folding and GC Content of Their 3′ UTRs

**Figure 5:**
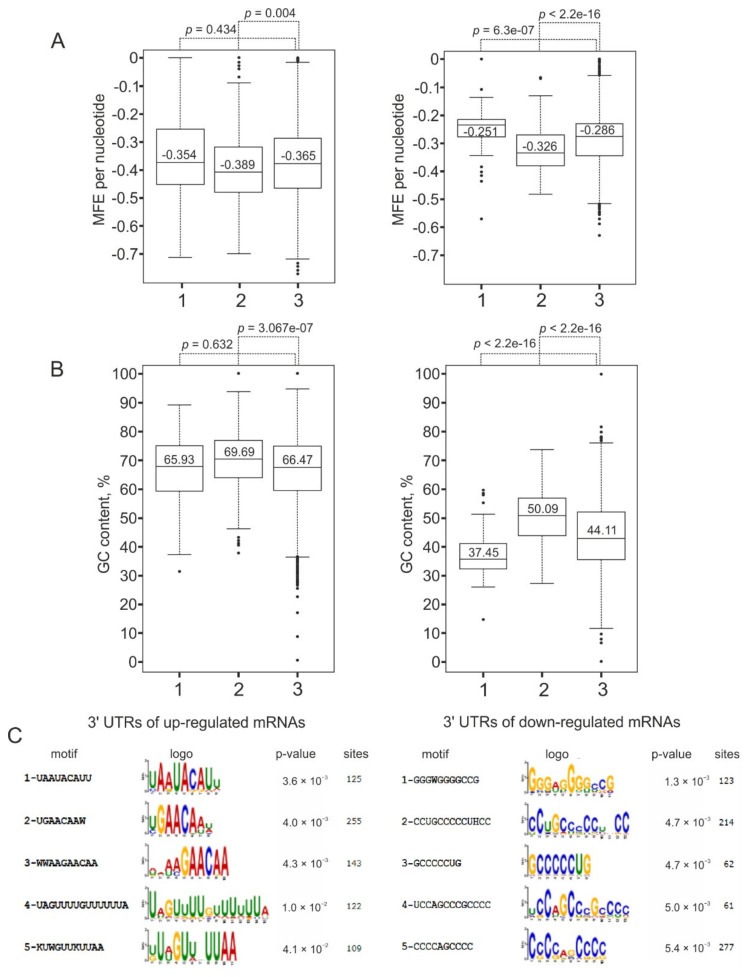
Sequence features of 5′ and 3′ UTRs of mRNAs of differentially expressed genes at the level of transcription, (t)DEGs, defined for uL5-deficient HEK293T cells. (**A**) Left, the distributions of minimum free energies (MFEs) calculated for 5′ UTRs of mRNAs of up-regulated (1) and down-regulated (2) (t)DEGs compared to that of MFEs determined for 5′ UTRs of all mRNAs of HEK293T cells (3), shown in the box and whisker diagrams; right, the same for the 3′ UTRs. (**B**) Left, GC contents calculated for 5′ UTRs of mRNAs of up-regulated (1) and down-regulated (2) (t)DEGs compared to those determined for 5′ UTRs of all mRNAs of HEK293T cells (3); right, the same for the 3′ UTRs of mRNAs. (**C**) Top 5 from the set of motifs (with lowest *p* values) identified in 3′ UTRs of mRNAs of up-regulated (left) and down-regulated (right) (t)DEGs. Column “sites” means a number of sites for the respective motif.

To find out if there are any structural similarities between mRNAs of up-regulated and down-regulated (t)DEGs, we analyzed the features of their UTRs. In this line, we performed a structural folding prediction and estimated the minimum free energy (MFE) of the 5′ and 3′ UTRs of the (t)DEG mRNA sequences using the RNAfold algorithm. The analysis revealed that the 3′ UTRs of mRNAs of down-regulated (t)DEGs were about 1.5-fold more structured (according to the values of MFE) compared to those of up-regulated ones (Figure 5A), whereas no significant differences were observed between their 5′ UTRs. We also compared the GC contents in the 5′ and 3′ UTRs of mRNAs of down-regulated and up-regulated (t)DEGs and found that the 3′ UTRs of down-regulated (t)DEG mRNAs have a higher GC content than those of up-regulated ones (Figure 5B). Again, no significant differences in the GC contents were found between the 5′ UTRs of mRNAs of down-regulated and up-regulated (t)DEGs. In addition, we analyzed the landscapes of the most common, over-represented motifs in the 3′ UTRs of mRNAs of up-regulated and down-regulated (t)DEGs and revealed that the 3′ UTRs of up-regulated (t)DEG mRNAs were enriched in AU-rich sequences, while those of down-regulated (t)DEG mRNAs contained preferably GC-rich ones (Figure 5C). All this suggests that under conditions of uL5 deficiency in cells, the rate of degradation of stable mRNAs with structured 3′ UTRs is elevated, while that of mRNAs with poorly structured 3′ UTRs is reduced, which leads to a decrease in the relative content of the former and vice versa, to an increase in that of the latter. Therefore, the structure of (t)DEG mRNA 3′ UTRs is more likely to play a role in the regulation of the expression of the respective genes at the post-transcriptional level rather than the transcriptional one.

No significant features were found in the structures of the GATE mRNA UTRs. Both up-regulated and down-regulated GATEs included both highly expressed genes (e.g., *POLR2A*, *RPLP1*, *RPL12* and *RPLP0*) and low expressed ones (e.g., *NPHP4*, *LPIN3*, *DTNB* and *SLC35A3*) (see baseMean column in Table S2). However, the average length of coding sequence (CDS) in up-regulated GATE mRNAs (2722 nucleotides) was significantly longer than that of CDS in human mRNAs (1278 nucleotides [21]), while this parameter for down-regulated GATE mRNAs was much less (947 nucleotides) (Table S8). This could be due to a lower frequency of events of the formation of 80S initiation complexes upon a deficiency of 60S subunits observed in uL5 knocked down cells compared to normal cells (Figure 1B), which, in turn, resulted in a significant decrease in the relative density of ribosomes per mRNA. The latter was not so critical for mRNAs with long CDSs allowing the placement of 5 or more ribosomes, since even with a multiple decrease in the number of ribosomes associated with them, they could remain bound to polysomes. In contrast, for mRNAs with short CDSs, such a decrease in the density of ribosomes should lead to an increase in the pool of mRNAs free of ribosomes, i.e., untranslated mRNAs. Thus, GATEs fell into sets of up-regulated and down-regulated genes depending on the length of the CDS in their mRNAs.

## 3. Discussion

By exploiting specific siRNAs, we induced a deficiency of the ribosomal protein uL5 in HEK293T cells and studied the landscapes of the total mRNAs and translating mRNAs. We showed that an approximately three-fold decrease in the uL5 content causes a significant deficiency of 60S subunits, resulting in a reduction in the level of 80S monosomes but without a noticeable diminution in the efficiency of translation in polysomes. Using the RNA-seq and polysome profiling followed by RNA-seq assays, we revealed differentially expressed genes for transcriptional and translational levels, (t)DEGs and (p)DEGs, between uL5 knocked down and control cells. Among transcriptionally activated (t)DEGs, there were genes predominantly related to rRNA processing, pre-mRNA splicing, translation, DNA repair and some others, whereas down-regulated (t)DEGs were mainly associated with the interactions involving membrane components. The structural analysis of (t)DEG mRNAs revealed increased and decreased GC contents in 3′ UTRs of mRNAs of down-regulated and up-regulated (t)DEGs, respectively. The distribution of minimum free energies for 3′ UTRs of mRNAs from these groups of genes was similar. By normalizing of results on differential gene expression analysis with the ribosome profiling followed by RNA-seq data to those of the analysis with the RNA-seq data, we identified genes with altered translation efficiency, GATEs, which were found to be associated with a wide range of cellular events.

Obviously, the deficiency of mature 60S subunits in cells with uL5 knockdown was caused by an insufficient amount of 5S RNPs in whose formation uL5 participates together with uL18. The shortage of 60S subunits, in turn, led to a reduction in the rate of formation of 80S initiation complexes, resulting from the joining of 48S pre-initiation complexes with 60S subunits. This follows from our data on a significant decrease in the content of the fraction of 80S monosomes comprising various types of complexes with a stoichiometric ratio of 80S ribosomes to mRNA, including 80S initiation ones. Although we did not observe a noticeable decrease in the level of polysomes, we found changes in the compositions of the total cellular mRNA and the fraction of translating mRNAs. These changes were undoubtedly caused by the cell’s reaction to the imbalance in uL5, which disrupted the normal state of the translational machinery, the efficiency of its operation and, ultimately, the productivity of protein synthesis. Therefore, the transcriptional activation of genes associated with rRNA processing and translation, which are present in the set of up-regulated (t)DEGs, is quite justified.

As far as can be judged from the structural features of 3′ UTRs of mRNAs of up-regulated and down-regulated (t)DEGs, the change in the translation initiation rate caused by a deficiency of 60S subunits somehow affects the stability of mRNAs. Since the initiation of translation of mRNAs with highly structured 3′ UTRs seems to be more difficult, as compared to that of mRNAs with poorly structured 3′ UTRs, the former mRNAs should be more susceptible to degradation than the latter ones. Indeed, according to the data reported in [22], in mammalian cells, mRNAs with GC-rich 3′ UTRs are generally less stable than mRNAs with AU-rich 3′ UTRs. Consequently, one can believe that in cells with a deficiency of uL5, the degradation of poorly translated mRNAs with a high GC content in their 3′ UTRs is enhanced compared to that in cells with the normal level of uL5. This means that to maintain an optimal balance between processes of transcription and protein synthesis upon a reduced uL5 content, cells rearrange their transcriptome by decreasing the stabilities of mRNAs with highly structured 3′ UTRs.

Besides, the effect of changing the rate of formation of 80S initiation complexes in uL5-deficient cells is also manifested in the size of mRNA CDSs of genes included in the sets of up-regulated and down-regulated GATEs. Obviously, with a decrease in the frequency of acts of translation initiation, mRNAs with short CDSs are less likely to participate in the initiation process than at the frequency of such acts in cells with the normal uL5 level. Therefore, the efficiency of translation of these mRNAs becomes decreased, which is supported by the data that down-regulated GATEs are genes whose mRNAs have short CDSs. At the same time, mRNAs with long CDSs, which are translated correspondingly longer than mRNAs with short CDSs, should remain in polysomes upon a reduction in the frequency of acts of translation initiation. Consequently, the relative share of these mRNAs should be increased compared to that of mRNAs with short CDSs, which is also confirmed by our finding that genes of mRNAs with long CDSs are up-regulated GATEs.

As mentioned in the Introduction, early studies on U2OS (human osteosarcoma) cells have shown that an increase in the content of uL5 leads to a decrease in the level and activity of the c-Myc proto-oncogene, presumably due to a decrease in the stability of its mRNA caused by the binding of a specific miRNA to the 3′ UTR of c-Myc mRNA [8,9]. Moreover, using RT-qPCR, a significant increase in the level of c-Myc mRNA itself was demonstrated in uL5-deficient U2OS, WI38 (human lung fibroblasts) and HEK293T cells [9]. However, we did not find any significant change in the level of c-Myc mRNA in uL5-deficient HEK293T cells, either when analyzing the RNA-seq data or when using RT-qPCR to determine its content. The reasons for the discrepancy between our data and those of the above study remain unclear, and this issue requires a special study.

The presence of genes *RPL12*, *RPLP0* and *RPLP1* encoding ribosomal proteins uL11, uL10 and P1, respectively, which are components of the 60S subunit P stalk in the set of down-regulated GATEs, is most likely related to the formation of this stalk at the final step of maturation of 60S subunits in the cytoplasm. It is well-known that it is at this step that ribosomal proteins uL10 and P1 are assembled into the pre-60S subunit [23,24], and, possibly, the uL11 protein does the same. Consequently, upon the insufficient production of pre-60S ribosomal subunits in cells with uL5 deficiency, an excess of these ribosomal proteins should accumulate in the cytoplasm. Given the high expression levels of *RPL12*, *RPLP0* and *RPLP1* genes in HEK293T cells (see Table S2), one could conclude that the amounts of excess proteins uL11, uL10 and P1 were quite significant in uL5 knocked down cells, which allowed the proteins to bind to their own coding mRNAs and thereby to reduce the levels of these mRNAs. A similar regulatory feedback mechanism has been found earlier for human genes encoding ribosomal proteins, such as eS26 [25], uS15 [26], eL29 [27] and uS3 [28], which have been shown to be able to bind with their own mRNAs in the cytoplasm or with their pre-mRNAs in the nucleus, inhibiting translation or splicing, respectively. It is quite possible that this mechanism takes place in the regulation of genes encoding uL11, uL10 and P1 as well.

To date, many studies have been carried out on the identification of abnormalities in gene expression in cells and organisms with haploinsufficiency of the ribosomal protein uL5, resulting from mutations in its gene, in order to understand the reason(s) for the progression of DBA in the chronic deficiency of this protein (see, e.g., Refs. [3,29,30]). In general, all these studies suggest that the reason is most likely not a dysregulation of some specific gene(s), but a decrease in the level of ribosomes, leading to an imbalance of translated mRNAs and, accordingly, a change in the translational profile of cells, which is crucial for hematopoietic progenitor ones. Our study with uL5 knocked down HEK293T cells, on the contrary, gives information not so much on the gene expression landscape typical of chronic uL5 deficiency, but on the landscape changes that appear when a deficiency of uL5 occurs, i.e., general changes in gene expression that can be caused by somatic mutations in the *RPL11* gene and initiate cancer. Such mutations in heterozygous variants, resulting in inactive forms of uL5 and found in human malignant neoplasms, have recently been reported in [31]. In this line, our data on the up-regulation of the expression of a large number of genes involved in rRNA processing, pre-mRNA splicing and translation, i.e., genes necessary for accelerated cell growth, can help in identifying pathways leading to the malignant transformation of cells in the event of irreparable uL5 deficiency. A comparative comprehensive analysis of transcriptomes and translatomes of differentiated cells with a chronic shortage of uL5 as DBA models and those of uL5 knocked down cells may facilitate the understanding of why a deficiency of this protein results in impaired lineage commitment in hematopoietic progenitor cells and malignant transformation in cells other than former ones.

Since the deficiency of uL5 leads to a decrease in the level of 60S subunits, it can be assumed that changes in gene expression similar to those found in this study will also occur upon the deficiency of other proteins classified as essential for the assembly of the 60S ribosomal subunit. Nevertheless, in addition to similarities, there may be differences in the changes as well because the uL5 and uL18 proteins are involved in the assembly process being bound to 5S rRNA, which distinguishes the mechanism of their participation in the 60S subunit biogenesis from that for many other ribosomal L-proteins. This is indicated by the data, showing that the loss of uL5 or uL18 does not lead to the distinct cell cycle arrest observed with the knockdown of other essential ribosomal proteins [16,32]. At the same time, there is no doubt that the deficiency of 60S subunits, regardless of what causes it, leads to a specific rearrangement of the landscape of cellular mRNAs, which can depend on the GC content in their 3′ UTRs, as shown in our study with L5-deficient cells.

Thus, the use of RNA-seq and polysome profiling followed by RNA-seq analyses to identify genes whose expression at transcriptional and translational levels is changed in uL5-deficient HEK293T cells allowed us to determine the ways by which cells restructure their transcriptome and translatome when the protein content is lowered and to reveal genes with altered translational efficiency. Our findings show that almost all the effects of the reduced level of uL5 on gene expression are mainly associated with a deficiency in the number of 60S subunits in cells, which inevitably leads to an insufficiency of ribosomes translating mRNAs. Therefore, it should be expected that the mechanisms of regulation of gene expression at the levels of transcription and translation are common for mammalian cells deficient in any ribosomal protein required for the assembly of functionally active ribosomal subunits. In general, the knowledge gained, together with the conclusions drawn on its basis, is of great importance for understanding changes in the physiological state of mammalian cells under appropriate conditions and for further research aimed at uncovering the cellular mechanisms leading to an increased risk for cancer.

## 4. Materials and Methods

### 4.1. Preparation of siRNAs, Cells Transfection, Collection of Cellular Lysates, Polysome Profiling and RNA Isolation

Oligoribonucleotides used as uL5 mRNA-specific siRNAs and control non-targeting siRNA (listed in Table S9) were prepared as described in [33]. HEK293T cells (CVCL_0063) were grown in 15 cm Petri dishes, transfected with siRNAs in four biological replicates, cultured, harvested and lysed according to the previously described procedures [33] with minor modifications. Briefly, 20 million of transfected cells were washed with ice-cold PBS containing 100 μg/mL of cycloheximide, collected in an Eppendorf tube and lysed in 800 μL of buffer 20 mM Tris-HCl (pH 7.5) containing 200 mM KCl, 15 mM MgCl_2_, 100 μg/mL of cycloheximide and 1% Triton-X100, and the lysate was cleared by centrifugation at 1500× *g* for 1 min at 4 °C. MTT test with transfected cells and the RT-qPCR analysis of uL5 mRNA content in these cells were performed as described in [33]. The knockdown of uL5 was confirmed by the Western blotting of lysate aliquots using specific rabbit polyclonal antibodies against uL5 (Proteintech, Rosemont, IL, USA, #16277-1-AP). Rabbit antibodies against GAPDH (Proteintech, #60004-1-Ig) were used as references. One quarter of the lysate (extracted from 5 million cells) was mixed with TRIzol Reagent (Ambion, Waltham, MA, USA) to isolate total cellular RNA, and the remaining three quarters (extracted from 15 million cells) were subjected to sucrose density gradient ultracentrifugation to generate a polysome profile, as described in [27] with minor modifications. Briefly, the extract was layered onto a 5 to 50% linear sucrose gradient in 50 mM buffer Tris–HCl (pH 7.5) containing 100 mM KCl and 12mM MgCl_2_ and centrifuged at 19,000 rpm for 17 h at 4 °C in a SW40 rotor. After the centrifugation, the gradients were fractioned through the flow cell of a Millichrom A-02 chromatograph (Econova, Novosibirsk, Russia) with monitoring of the UV absorption profile at 260 nm and collecting fractions on ice. Four-fifths volumes of each of the polysome-containing gradient fractions were pooled, and 0.7 volumes of ice-cold ethanol were added to the resulting fraction in the presence of 20 mM MgCl_2_, followed by centrifugation at 14,000× *g* for 30 min at 4 °C. The precipitate was dissolved in water, followed by the addition of TRIzol Reagent. Samples of total cellular RNA and RNA from polysomes were extracted from the respective TRIzol Reagent-containing mixtures according to the manufacturer’s protocol. A fifth of each gradient fraction was Western blotted using rabbit polyclonal antibodies specific for ribosomal proteins uL5, eL28 (Thermo Fisher Scientific, Waltham, MA, USA, PA5-101387) and eS26 (Proteintech, #14909-1-AP), as above.

### 4.2. DNA Libraries Preparation and NGS

The resulting RNA samples were quality-checked with the Bioanalyzer 2100 (Agilent, Santa Clara, CA, USA) using the RNA6000Pico kit. DNA libraries were prepared using the MGIEasy RNA Directional Library Prep Set (MGI Tech, Shenzhen, China) according to the manufacturer’s instructions and subjected to NGS on the MGISEQ-2000 platform utilizing the 2 × 100 PE sequencing mode.

### 4.3. Raw NGS Data Processing

Raw reads in fastq formats were assessed for quality using FastQC (v. 0.11.9) (www.bioinformatics.babraham.ac.uk/projects/fastqc/) (accessed on 9 February 2021) and MultiQC (v. 1.9) [33] tools and subjected to quality filtering (Trimmomatic 0.39) [34] and adapter trimming (cutadapt) using sequences provided by the manufacturer. The filtered reads were also assessed for quality and subjected to the mapping procedure with the STAR RNA aligner tool (v. 2.7.3) [35] using the hg38 reference human genome and the Ensembl annotation (release 102). The quality of the obtained BAM files was checked using the QualimapTool (v.2.2) [36]. All the RNA-seq read data were submitted to the GenBank under the study accessions PRJNA765729.

### 4.4. Bioinformatics Analysis of the Processed NGS Data

A table with raw read counts assigned to each gene (counts table) was generated from data of the RNA-seq and polysome profiling followed by RNA-seq assays with the application of the Rsubread package (v. 2.4.0) [37] using the featureCounts function with the GTF file (ensembl release 102) as an annotation in the reversely stranded mode. The biomaRt package (v. 2.46.0) [38] was utilized for the annotation of genes with their HGNC symbol, entrez id and description. Based on the counts table, analysis for (t)DEGs was performed using RNA-seq data obtained with total RNA samples from cells knocked down of uL5 and control cells treated by non-targeting siRNA with the application of the DESeq2 (v. 1.30.0) package [39] with default parameters. In the analysis, the apeglm algorithm was exploited to shrink LFC values. Analysis for (p)DEGs was carried out similarly using RNA-seq data obtained with RNA samples from the respective polysome fractions. For the selection of DEGs, the p.adj cutoff was assigned to 0.05, and the absolute value of shrunken LFC cutoff was assigned to 0.322 (i.e., only those changes in gene expression levels were taken into account, which were more than 25% of the levels in control cells). GATEs were identified by differential gene expression analysis with the RNA-seq and polysome profiling with followed RNA-seq data using DESeq2, as described in the systemPipeR package vignette. The plot illustrating the results of the analysis was built using the ggplot2 package. For plotting, the mean values for the RNA-seq data and for the polysome profiling followed by RNA-seq data were calculated, and after adding 1 pseudo-count to each mean value, a log2-transformation was performed. Genes with baseMean value < 100 were cut off, and GATEs were labeled by the HGNC symbols. The PCA plots were visualized with DESeq2 internal functions. The pathway enrichment analysis was performed using the ReactomePA package (v. 1.34.0).

### 4.5. Validation of NGS-Derived Results Using RT-qPCR

Reverse transcription (RT) was carried out using 2.5 µg of RNA samples isolated from aliquots of the respective cell lysates or pooled polysome gradient fractions as indicated above, 100 pmol of random hexamer primer and 20 U of MMLV reverse transcriptase according to [40]. The resulting cDNA was then used for qPCR analysis, performed as described in [40] using appropriate gene-specific primers (Table S10). The experiments were performed in four biological replicates. Relative levels of gene expression were quantified using the integrated LightCycler 96 (Roche, Basel, Switzerland) software using *GAPDH* and *TUBB* gene expression levels as references.

### 4.6. Analysis of the Parameters of mRNA Structures

The sequences of mRNA 5′ UTRs and 3′ UTRs for the subset of (t)DEGs were obtained with the biomaRt (v. 2.46.0) [38]. To extract the UTR sequences, only the canonical transcripts for each gene were used (according to the Ensembl annotation). For these sequences, the GC content values were calculated using the Biostrings package (v. 2.58.0) [41], and the MFE values were estimated using the LncFinder package (v. 1.1.4) [42] and RNAFold programs from the ViennaRNA package (v. 2.4.18) [43]. The rude MFE value per nucleotide was calculated by the division of the MFE value by the UTR length. The plots were created utilizing the ggplot2 package (v. 3.3.2) [44]. The *p* values for comparing means were calculated using the Student’s T-test. The motif enrichment analysis was performed using the STREME application from the MEME Suite (v.5.3.2) [45], with the (t)DEG mRNA UTR sequences as an input and the mRNA UTR sequences of all genes extracted from the Ensembl database (only canonical transcripts) as a control, with default parameters.

## Data Availability

RNA-seq data were submitted to the GenBank under the study accessions PRJNA765729.

## Supplementary Materials

The following are available online at https://www.mdpi.com/article/10.3390/ijms222413485/s1.

## Abbreviations

CDS	coding sequence
DBA	Diamond-Blackfan anemia
(p)DEG	differentially expressed gene at translational level
(t)DEG	differentially expressed gene at transcriptional level
ECM	extracellular matrix
GATE	gene with altered translational efficiency
L1CAM	L1 family of cell adhesion molecules
LFC	Log2 Fold Change
MFE	minimum free energy
NGS	next generation sequencing
PCA	principal component analysis
RNA-seq	high-throughput RNA sequencing
