# Supplementary material for: Deficiency of the Ribosomal Protein uL5 Leads to Significant Rearrangements of the Transcriptional and Translational Landscapes in Mammalian Cells
Source: Int J Mol Sci. 2021 Dec 15;22(24):13485. doi: 10.3390/ijms222413485 (PMC8706191; doi:10.3390/ijms222413485)
Supplement: Supplementary file 1 [file ijms-22-13485-s001.zip › Supplementary Materials.pdf]

Supplementary Materials

Figures

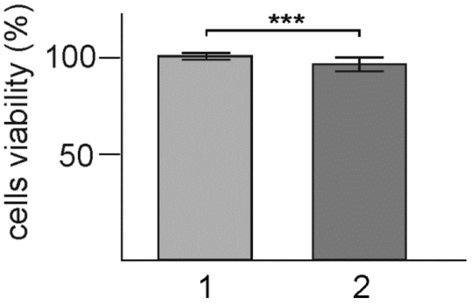

Figure S1. Histogram showing the viability of HEK293T cells according to the MTT test in six biological replicates, performed 48 h after transfection with non-targeting siRNA (1) or uL5 mRNA-specific siRNAs (2). Data are presented as mean values (%) ± SEM (*** p < 0.001, Mann-Whitney test).

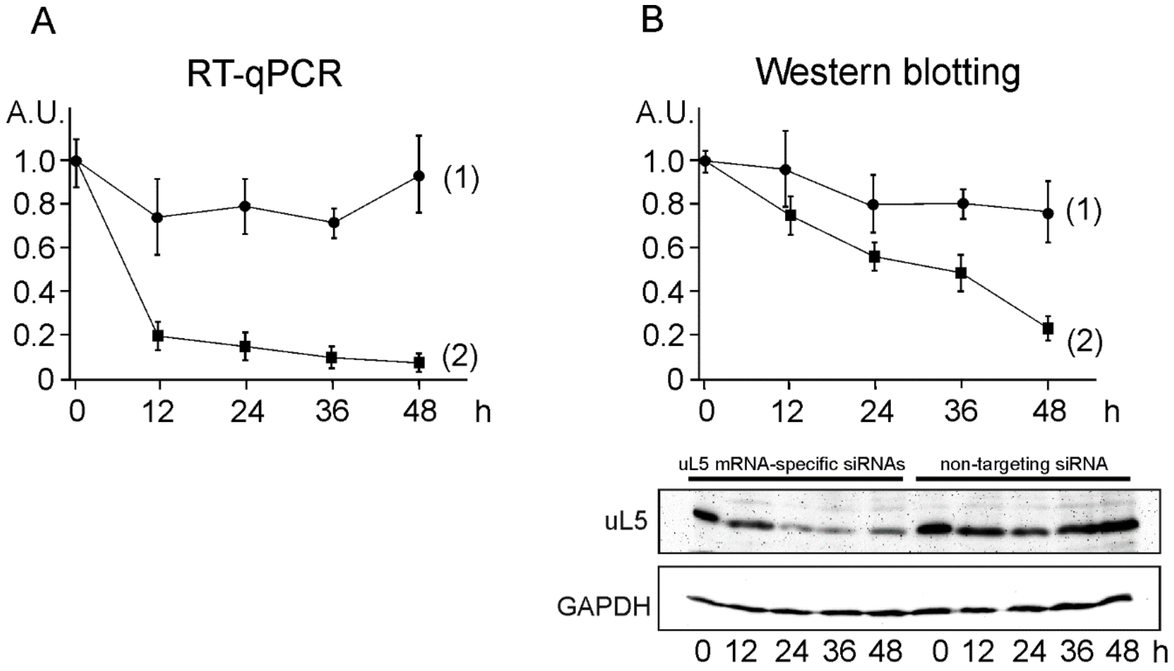

Figure S2. Time-course changes in the levels of ribosomal protein uL5 and its mRNA in transfected HEK293T cells. (A) Levels of uL5 mRNA normalized to GAPDH mRNA levels in cells transfected with non-targeting siRNA (1) or uL5 mRNA-specific siRNAs (2), determined at certain time intervals after transfection using RT-qPCR. The data are presented as the mean of arbitrary units (A.U.) ± SEM from 3 biological replicates. (B) Top panel, levels of uL5 normalized to those of GAPDH in cells transfected with non-targeting siRNA (1) or uL5 mRNA-specific siRNAs (2), as quantified by the densitometry of images of western blotting with specific antibodies performed at certain time intervals after transfection (using the VersaDoc imaging system (Bio-Rad) and Quantity One software). The data are presented as the mean of arbitrary units (A.U.) ± SEM from 3 biological replicates. Bottom panels, representative western blotting images showing uL5 and GAPDH levels in cells at certain time intervals after transfection with uL5 mRNA-specific siRNAs or non-targeting siRNA.

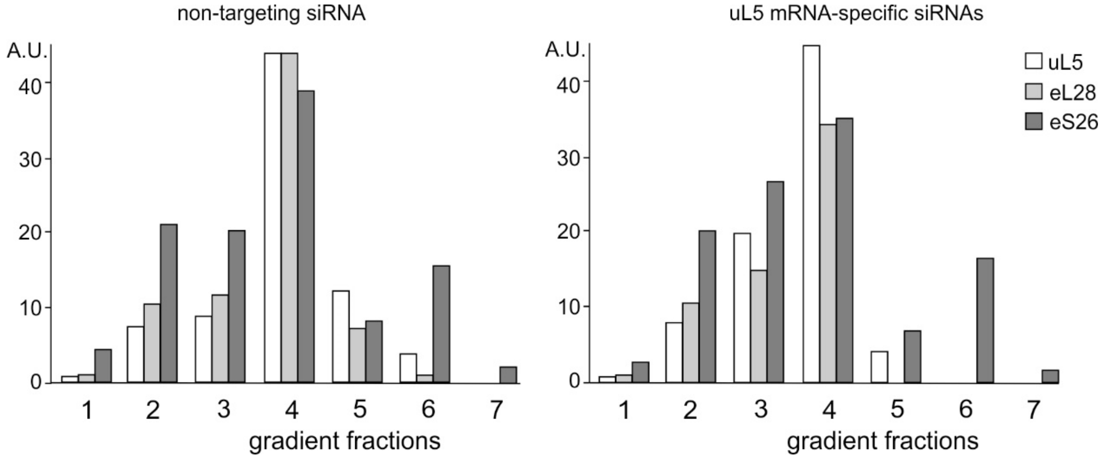

Figure S3. Diagrams of densitometric analysis of Western blot data presented in Figure 1B, showing relative contents of ribosomal proteins uL5, eL28 and eS26 in arbitrary units (A.U.) in gradient fractions of polysome profiles from cytosolic extracts of HEK293T cells treated with non-targeting siRNA and uL5-deficient cells. The relative error of the measurements was ~ 15%. The relative content of eL28 in fractions 4 and 5 corresponds to the total relative content of 80S monosomes and free 60S subunits in these fractions. Since the content of 60S subunits is equivalent to the content of 40S subunits in 80S ribosomes, the content of eS26 should also be equivalent to the content of eL28 in 80S monosomes and in polysomes where there are no free 40S and 60S subunits. This means that the ratio between the relative contents of eS26 and eL28 in the polysome fraction (fraction 2) should also be preserved for 80S monosomes contained in fractions 4 and 5. Thus, taking into account the value of the relative content of eL28 in fractions 4 and 5 and the value of the ratio of the relative contents of eL28 and eS26 in fraction 2, the share of 80S monosomes in fractions 4 and 5 can be calculated. The share of free 60S subunits in these fractions can be determined by subtracting the share of eL28 in 80S monosomes contained in fractions 4 and 5 from total relative content of the protein in these fractions. The ratio between the values of the relative content of eL28, attributable to the share of free 60S subunits in fractions 4 and 5, and of the relative content of eS26 in fraction 6, corresponding to 40S subunits, gives the ratio between free 60S and 40S subunits. As a result of the calculations, the 60S/40S ratio was found to be 1.7 for cells treated with non-targeting siRNA and 0.74 for uL5-deficient cells.

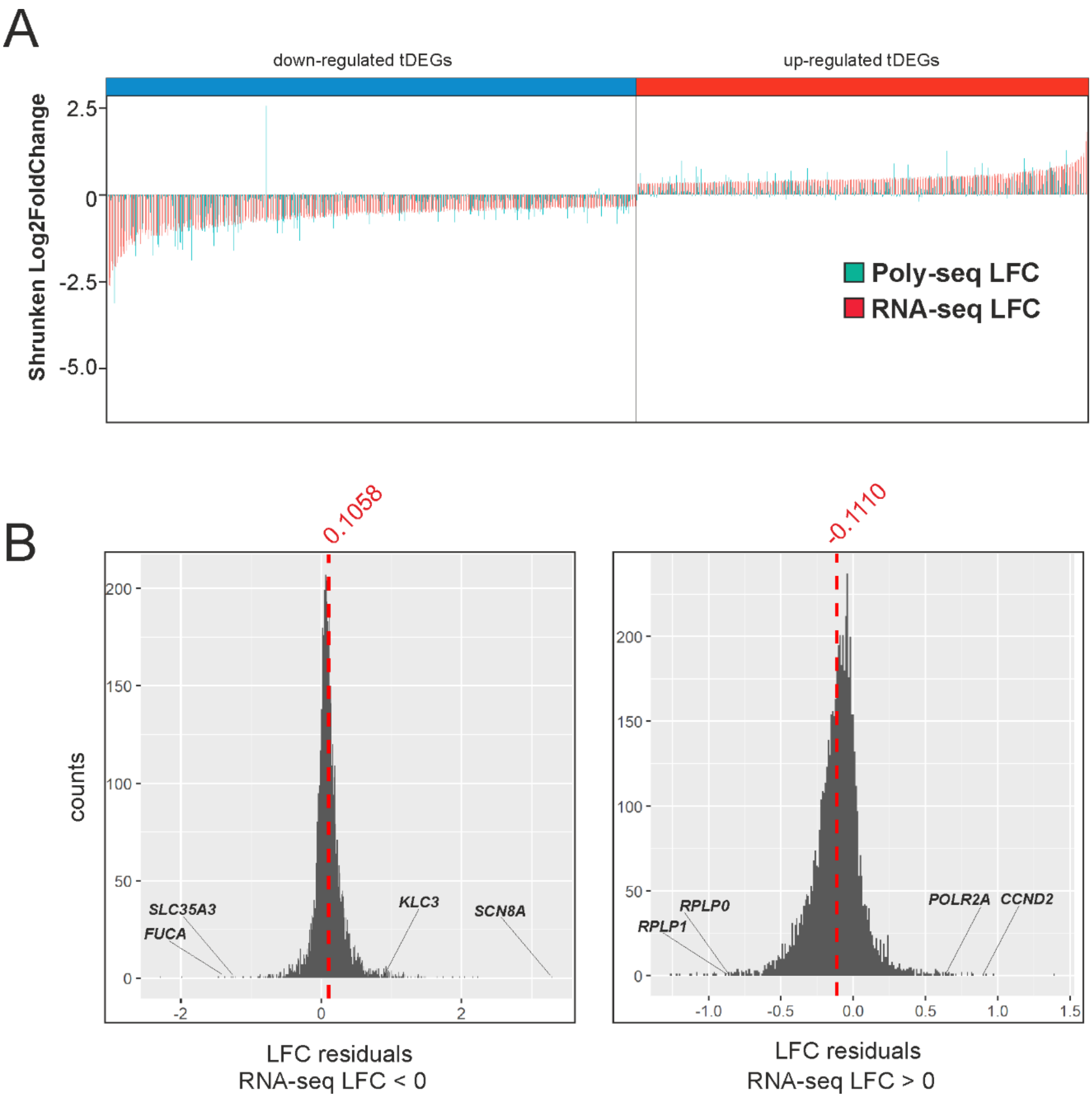

Figure S4. A comparative analysis of the RNA-seq data and the Poly-seq data. (A) The barplots of LFC values for sets of down-regulated and up-regulated (t)DEGs. For both sets of (t)DEGs, LFC values estimated based on the RNA-seq data and the Poly-seq data are presented (for the Poly-seq data, LFC values are given regardless of the p.adj threshold). Red bars correspond to LFC values estimated for (t)DEGs from the RNA-seq data, green bars correspond to LFC values estimated for (t)DEGs from the Poly-seq data. (B) The distribution of LFC residuals calculated from LFC values estimated based on the RNA-seq data and the Poly-seq data. The calculation was performed by a formula: LFC(res) = LFC(ps) – LFC(rs), where rs = RNA-seq, ps = Poly-seq. The distribution plots were created for genes with LFC(rs) < 0 (left panel) and LFC(rs) > 0 (right panel). The red dashed lines correspond to the mean of LFC residuals. Several pronounced genes predicted as GATEs are labeled.

Table S1. Basic characteristics of the obtained cDNA libraries and mapped reads (the Excel table)

Table S2. Results of the DESeq2 analysis of NGS data (the Excel table)

Table S3. A set of transcriptionally differentially expressed genes (designated as (t)DEGs) between uL5 knocked down and control HEK293T cells (the Excel table)

Table S4. A set of translationally differentially expressed genes (designated as (p)DEGs) between uL5 knocked down and control HEK293T cells (the Excel table)

Table S5. A set of genes with altered translation efficiency (designated as GATEs) in uL5 knocked down HEK293T cells compared to control cells (the Excel table)

Table S6. Reactome pathway analysis results for down- regulated (t)DEGs (the Excel table)

Table S7. Reactome pathway analysis results for up-regulated (t)DEGs (the Excel table)

Table S8. Evaluation of the mean CDS length for up-regulated and down-regulated GATEs (the Excel table)

Table S9. Sequences of siRNAs used in this work.

**Duplex number and oligonucleotide name**	**Sequence**
I. uL5 mRNA-specific-sense	5′- ggaacuucgcauccgcaaa**dTdT**-3′
I. uL5 mRNA-specific-antisense	5′-**phosphate**-uuugcggaugcgaaguucc**dCdT**-3′
II. uL5 mRNA-specific-sense	5′-aagcauugguaucuacggccu**dTdT**-3′
II. uL5 mRNA-specific-antisense	5′-**phosphate**-aggccguagauaccaaugcuu**dTdT**-3′
III. Control-sense	5′-uucuccgaacgugucacgu**dTdT**-3′
III. Control-antisense	5′- **phosphate**-acgugacacguucggagaa**dTdT**-3′

Table S10. The set of oligonucleotides used for RT-qPCR.

**Gene**	**Forward primer**	**Reverse primer**
RPL11	5′- gtctaaaggtgcgggagtat -3′	5′- tttgctgattctgtgtttggc -3′
RPLP0	5′- gtggagacggattacacctt-3′	5′- tcttccttggcttcaacctt-3′
KLC3	5′- caacaacttggctgtcctct-3′	5′- ctcaaacttgccctggttct-3′
JUN	5′-gaccttctatgacggatgccc-3′	5′-gttgctggactggattatca-3′
RPL29	5′-cgacttgcctacattgcc-3′	5′-cctgagctggaactgaag-3′
TUBB	5′-tggtggattctagaacctgga-3′	5′-ctgccccagactgaccaaat-3′
GAPDH	5′-gtgaaccatgagaagtatgacaac -3′	5′-catgagtccttccacgatacc -3′
